# Supplementary material for: Wood Degradation by Fomitiporia mediterranea M. Fischer: Exploring Fungal Adaptation Using Metabolomic Networking
Source: J Fungi (Basel). 2023 Apr 30;9(5):536. doi: 10.3390/jof9050536 (PMC10218912; doi:10.3390/jof9050536)
Supplement: Supplementary file 1 [file jof-09-00536-s001.zip › Supp_Figures_Schilling_et_al.pdf]

Journal of
Fungi

MDPI

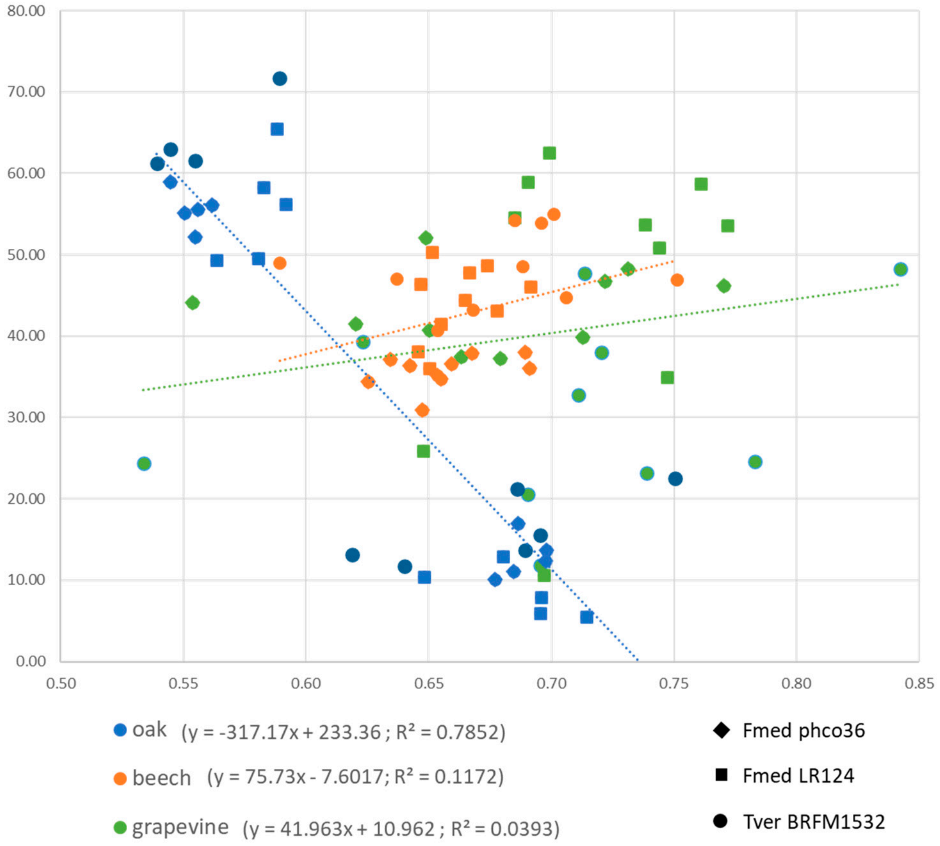

Figure S1. Wood blocks mass losses for each wood species after exposure to fungal degradation by the three strains Fmed phco36, Fmed LR124 and Tver BRFM1532, as a function of their initial density calculated on dry weight. Trend line equations and correlation coefficient (R^2) are given for each wood species exposed to all fungal strains taken together.

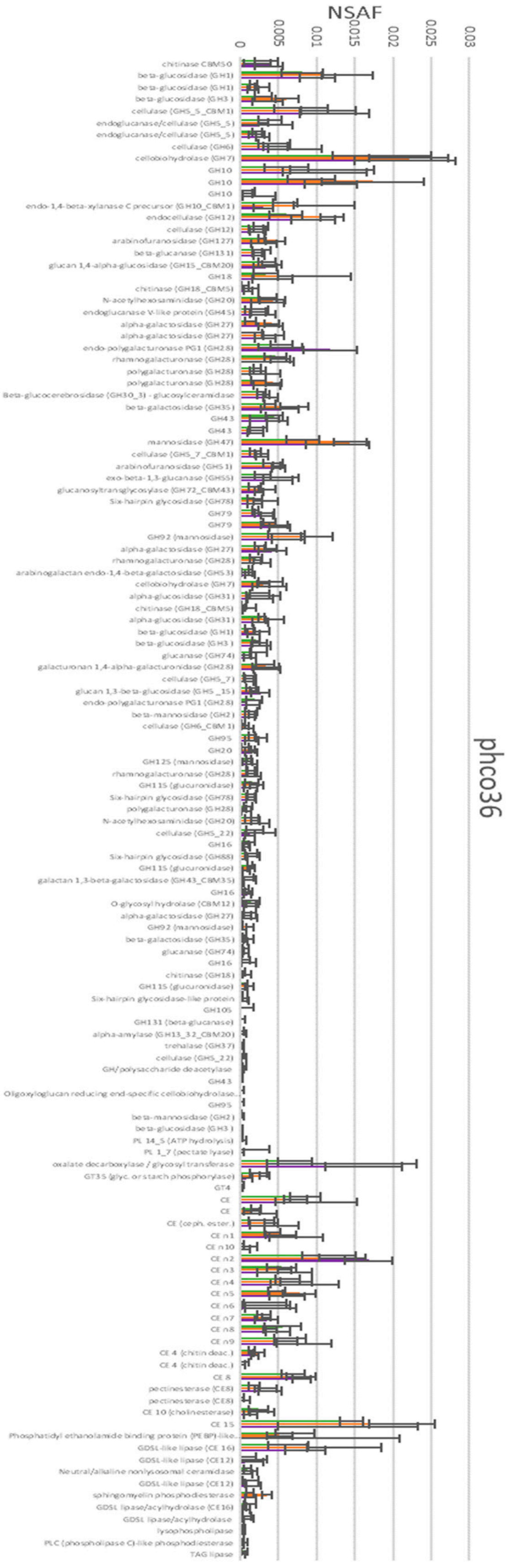
NSAF
0
0.005
0.01
0.015
0.02
0.025
0.03
phco36

(a)

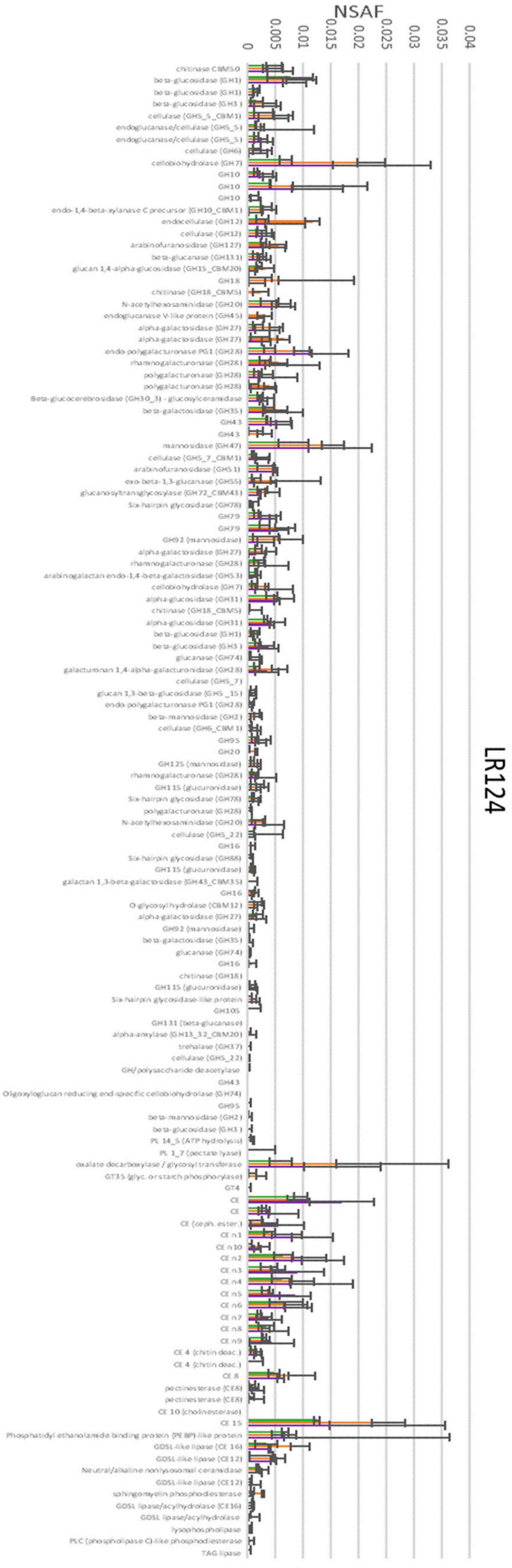
NSAF
0
0.005
0.01
0.015
0.02
0.025
0.03
0.035
0.04
LR124

(b)

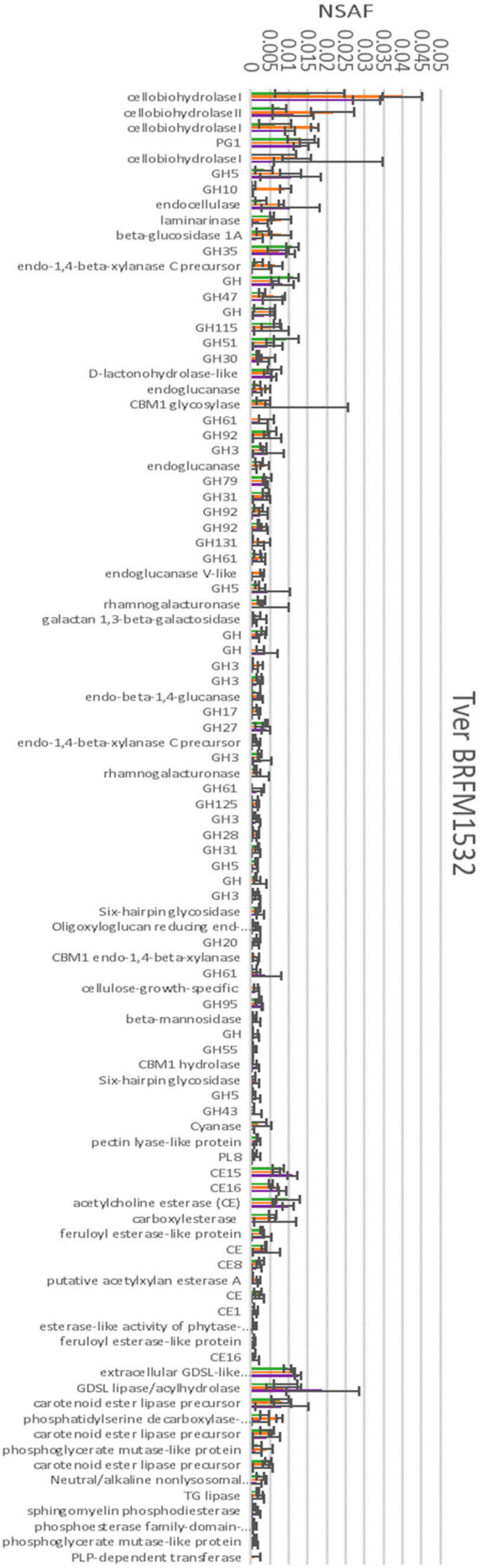
NSAF
0
0.005
0.01
0.015
0.02
0.025
0.03
0.035
0.04
0.045
0.05
cellobiohydrolase I
cellobiohydrolase II
cellobiohydrolase I
PG1
cellobiohydrolase I
GH5
GH10
endocellulase
laminarinase
beta-glucosidase 1A
GH35
endo-1,4-beta-xylanase C precursor
GH
GH47
GH
GH115
GH51
GH30
D-lactonohydrolase-like
endoglucanase
CBM1 glycosylase
GH61
GH92
GH3
endoglucanase
GH79
GH31
GH92
GH92
GH131
GH61
endoglucanase V-like
GH5
rhamnogalacturonase
galactan 1,3-beta-galactosidase
GH
GH
GH3
GH3
endo-beta-1,4-glucanase
GH17
GH27
endo-1,4-beta-xylanase C precursor
GH3
rhamnogalacturonase
GH61
GH125
GH3
GH28
GH31
GH5
GH
GH3
Six-hairpin glycosidase
Oligoxyloglucan reducing end-...
GH20
CBM1 endo-1,4-beta-xylanase
GH61
cellulose-growth-specific
GH95
beta-mannosidase
GH
GH55
CBM1 hydrolase
Six-hairpin glycosidase
GH5
GH43
Cyanase
pectin lyase-like protein
PL8
CE15
CE16
acetylcholine esterase (CE)
carboxylesterase
feruloyl esterase-like protein
CE
CE8
putative acetylxylan esterase A
CE
CE1
esterase-like activity of phytase-...
feruloyl esterase-like protein
CE16
extracellular GDSL-like...
GDSL lipase/acylhydrolase
carotenoid ester lipase precursor
phosphatidylserine decarboxylase-...
carotenoid ester lipase precursor
phosphoglycerate mutase-like protein
carotenoid ester lipase precursor
Neutral/alkaline nonlysosomal...
TG lipase
sphingomyelin phosphodiesterase
phosphoesterase family-domain-...
phosphoglycerate mutase-like protein
PLP-dependent transferase
Tver BRFM1532

(c)

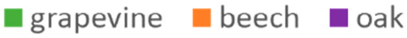

Figure S2. Relative abundancies (based on NSAF values) of CAZymes secreted by **(a)** Fmed phco36, **(b)** Fmed LR124 or **(c)** Tver BRFM1532 on grapevine, beech or oak wood at 4 months of culture. Error bars represent the standard mean error between triplicates.

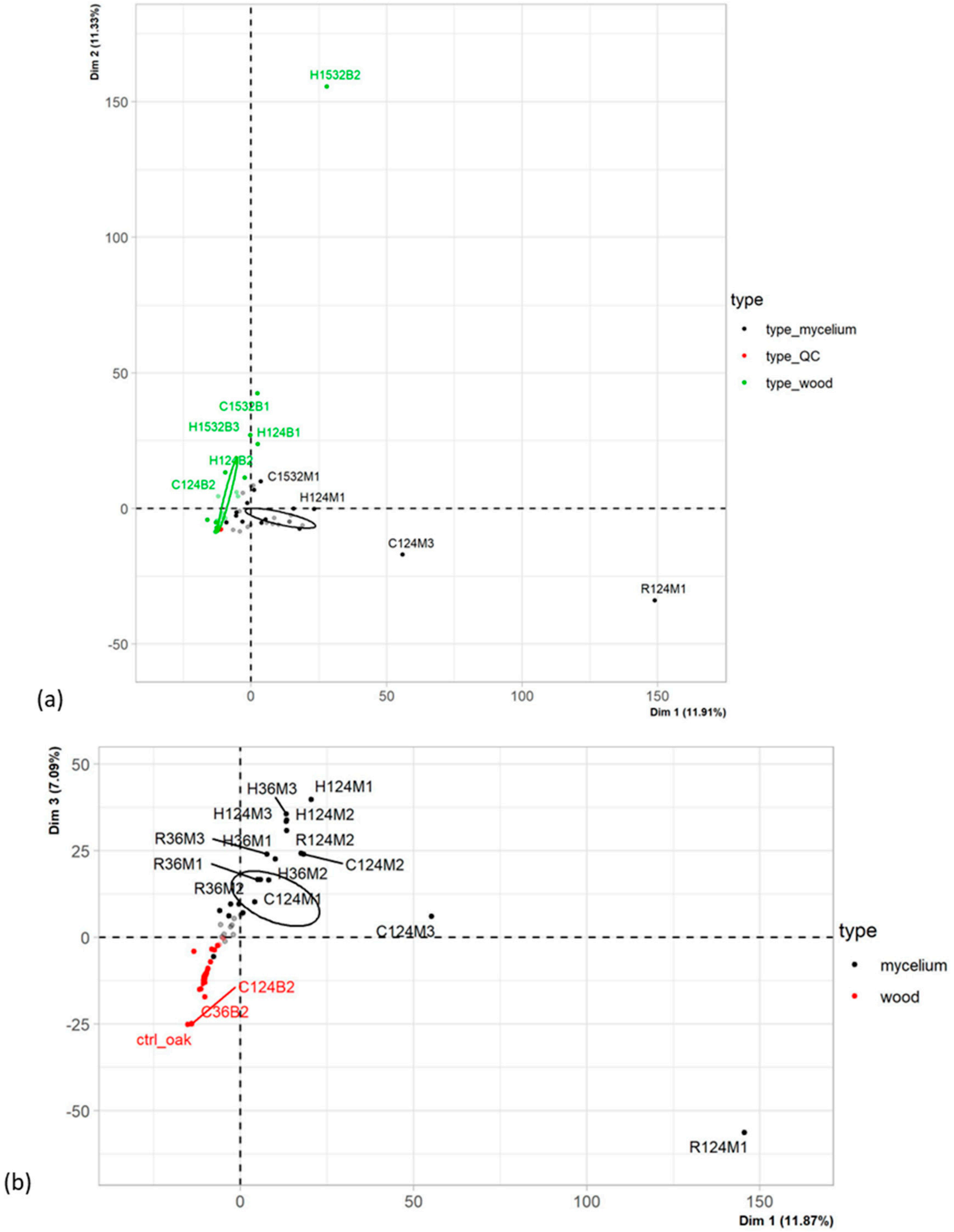

Figure S3 PCA analysis on all samples (a) with Quality Control (QC) samples on dimensions 1 and 2, (b) without QC on dimensions 1 and 3. Colors are given according to sample type (green (a) or red (b) = mycelia extracts from Fmed phco36 mycelia grown on grapevine ("R36M*X*"), beech ("H36M*X*") or oak("C36M*X*") wood); or red from Fmed LR124 mycelia grown on the three woods (resp. "R124M*X*", "H124M*X*" and "C124M*X*"), from Tver BRFM1532 mycelia grown on the three woods (resp. "R1532M*X*", "H1532M*X*" and "C1532M*X*"), or from Fmed phco36 ("ctrl36"), LR124 ("ctrl124") or Tver BRFM1532 ("ctrl1532") mycelia grown on malt-agar media without wood; red (a) = Quality Control ("QCMDS*X*"); black (a and b) = wood extracts from grapevine wood exposed to Fmed phco36 ("R36B*X*"), Fmed LR124 ("R124B*X*") or Tver BRFM1532 ("R1532B*X*"), from beech wood exposed to the three strains (resp. "H36B*X*", "H124B*X*", "H1532B*X*"), from oak wood exposed to the three strains (resp. "C36B*X*", "C124B*X*", "C1532B*X*"), or from grapevine, beech or oak control woods (resp. "ctrl_grapevine", "ctrl_beech" and "ctrl_oak"). For all sample names "*X*" refers to each biological repetition for wood and mycelia extracts, and to technical replicate for quality controls and blanks.

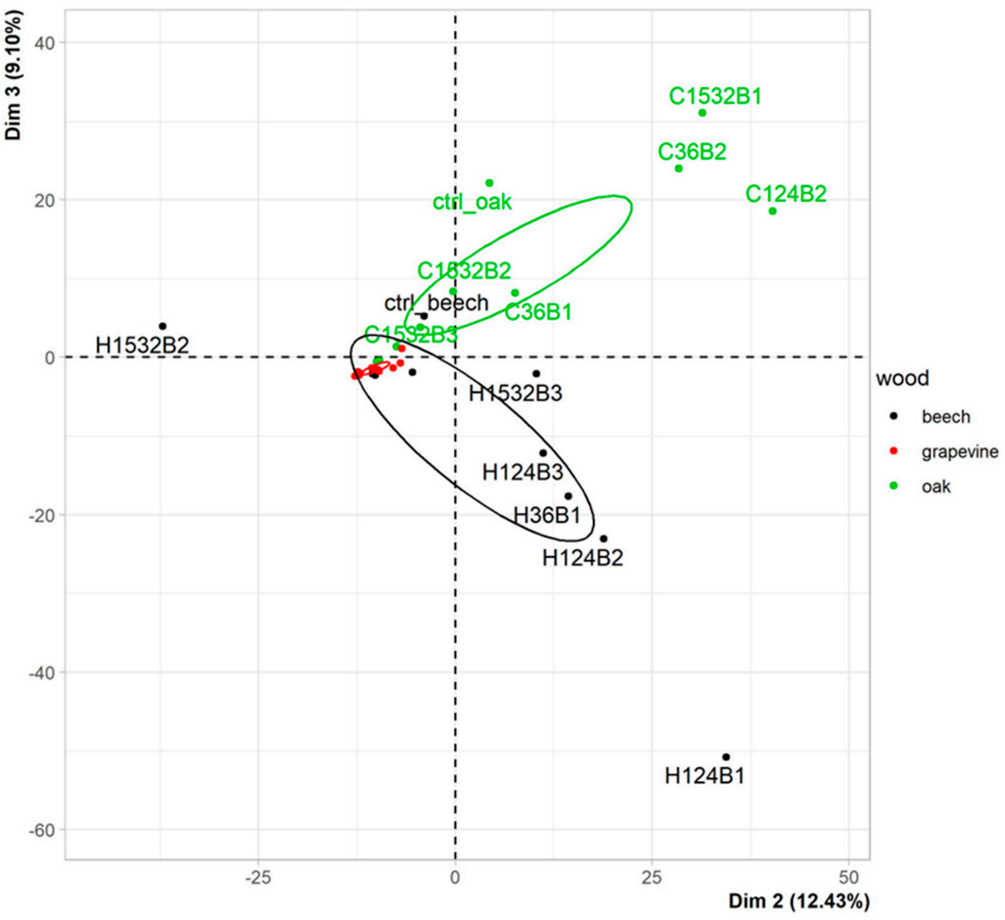

Figure S4. Dimensions 2 and 3 of PCA analysis on wood extracts. For sample names see Supplementary Material Figure S1 caption.

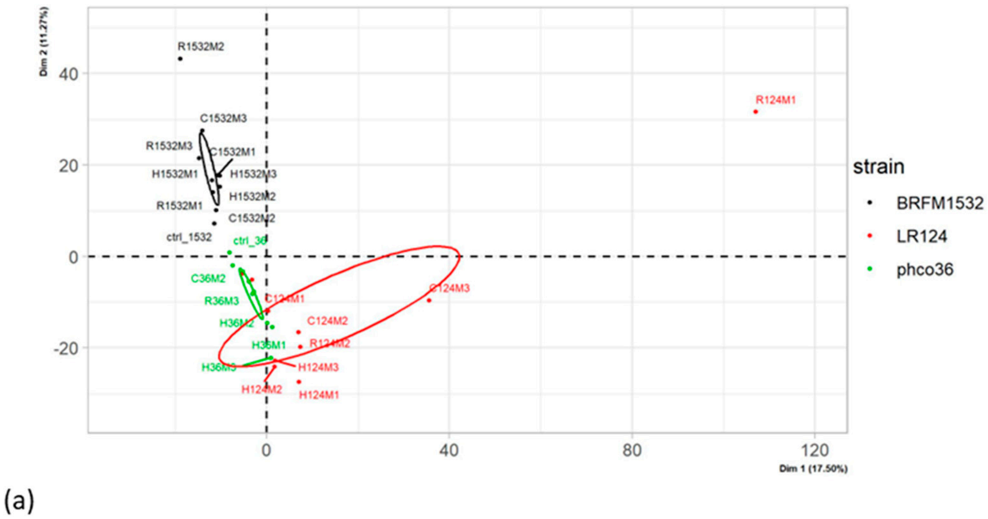

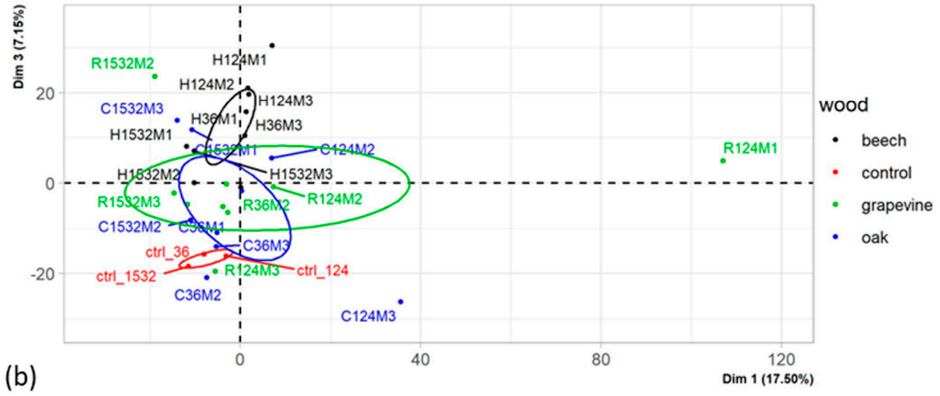

Figure S5. PCA analysis on mycelium extracts on dimensions (a) 1, 2 and (b) 1 and 3. For sample names see Supplementary Material Figure S1 caption.

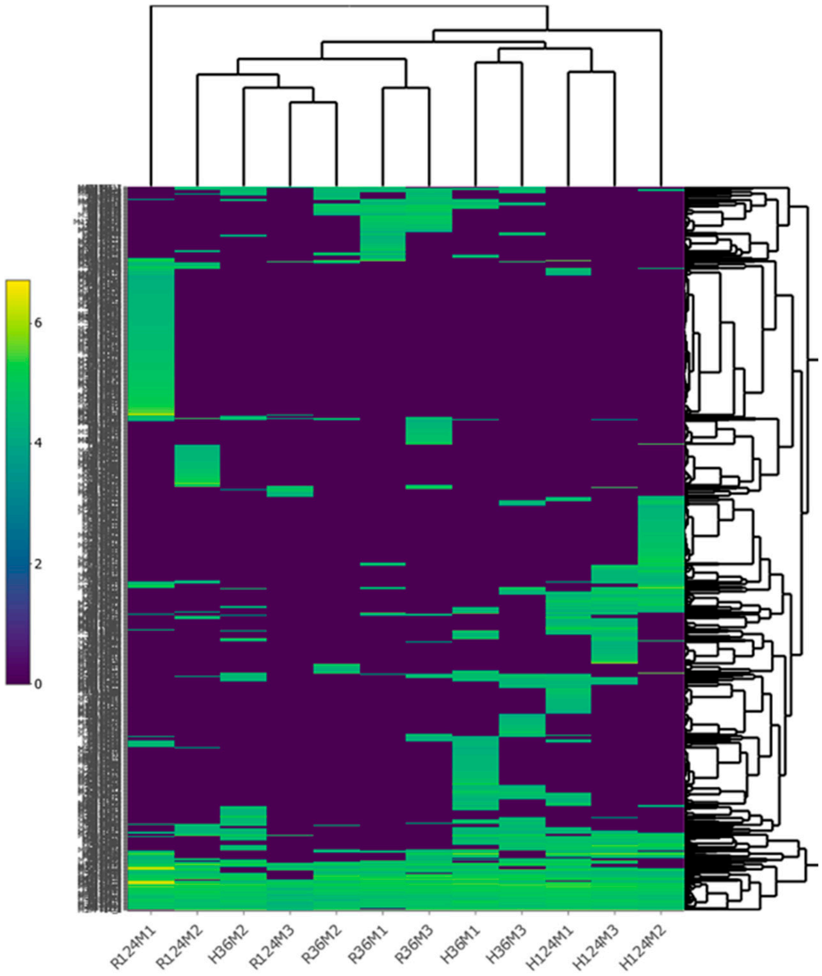
6
4
2
0
R124M1
R124M2
H36M2
R124M3
R36M2
R36M1
R36M3
H36M1
H36M3
H124M1
H124M3
H124M2

(a)

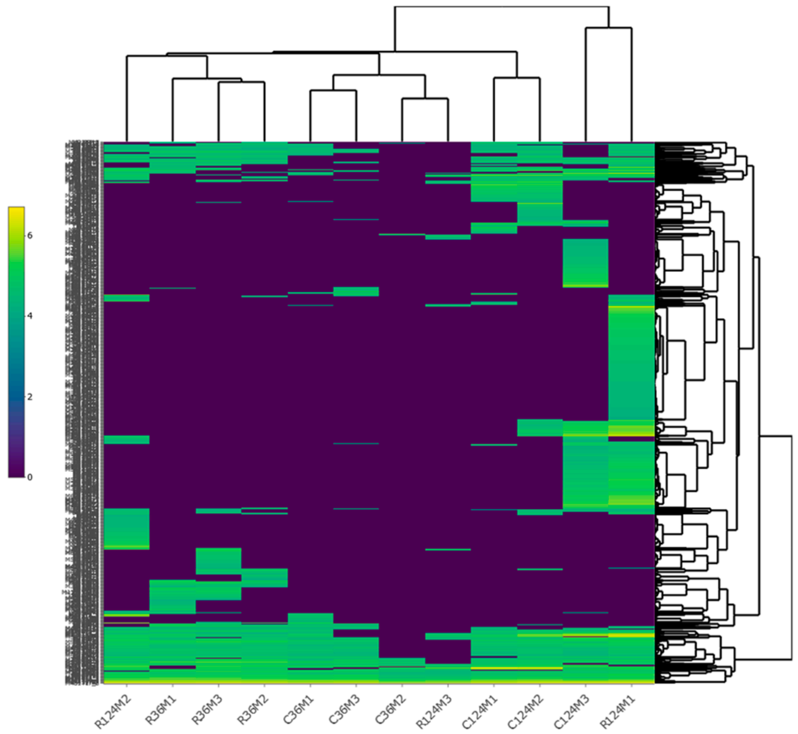

(b)

Figure S6. Log10 relative abundancies of MS features significantly different between Fmed mycelia grown with **(a)** grapevine or beech wood (851 features) and with **(b)** grapevine or oak wood (796 features). (For sample names see Supplementary Material Figure S1 caption.).

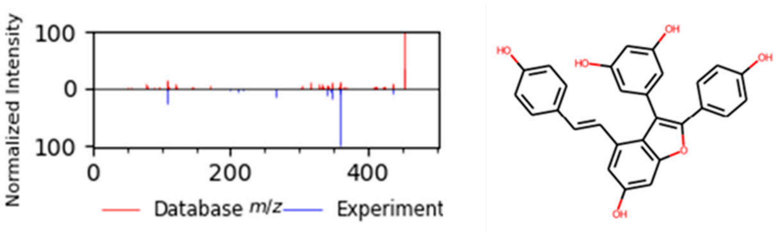

Figure S7. MS/MS spectra of feature annotated as compound 1 with *m/z* 453.133 (Experiment) compared to spectra from ISDB (*in-silico*) database (*m/z* 453.133), and *in-silico* proposed structure (ISDB - ©GNPS). Cosine score was 0.82.

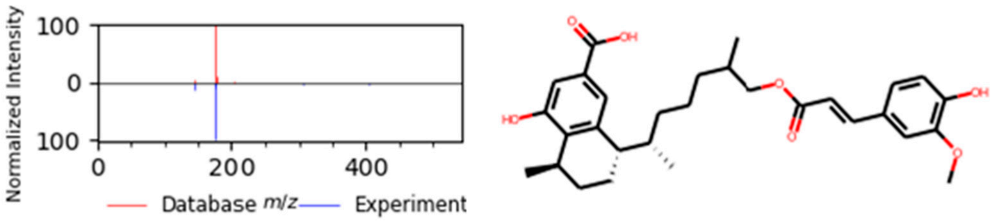

Figure S8. MS/MS spectra of feature with *m/z* 491.373 (Experiment) compared to KU036-11-7 M+H (*m/z* 511.268) spectra from GNPS-LIBRARY database, and structure of the standard (©GNPS). Cosine score as analogs was 0.82.

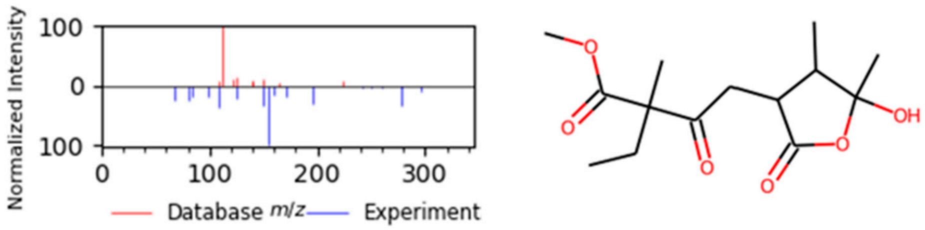

Figure S9. MS/MS spectra of feature with *m/z* 348.275 (Experiment) compared to methyl 2-ethyl-4-[(3R,4R,5S)-5-hydroxy-4,5-dimethyl-2-oxooxolan-3-yl]-2-methyl-3-oxobutanoate (*m/z* 304.176) spectra from database, and structure of the standard (©GNPS). Cosine score as analogs was 0.59.

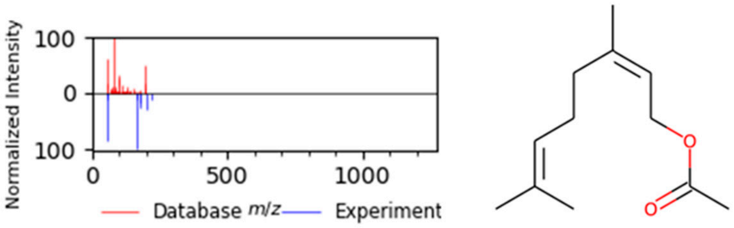

Figure S10. MS/MS spectra of feature with *m/z* 277.180 (Experiment) compared to nerylacetate (*m/z* 197.154) spectra from database, and structure of the standard (©GNPS). Cosine score as analogs was 0.80.

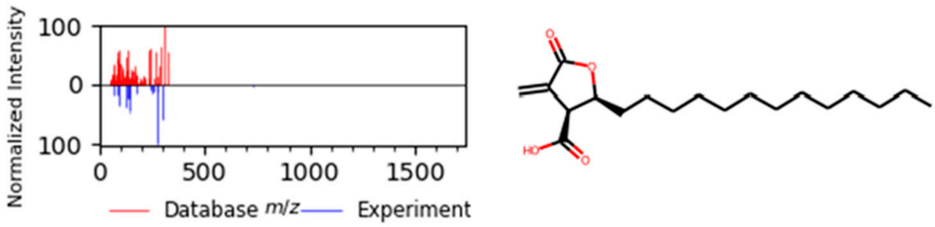

Figure S11. MS/MS spectra of feature with *m/z* 330.264 in cluster 69 (Experiment) compared to spectra from *allo*-protolichesterinic acid [M+NH4] database (*m/z* 342.364), and *allo*-protolichesterinic acid structure (ISDB - ©GNPS). Cosine score as analogs was 0.68.

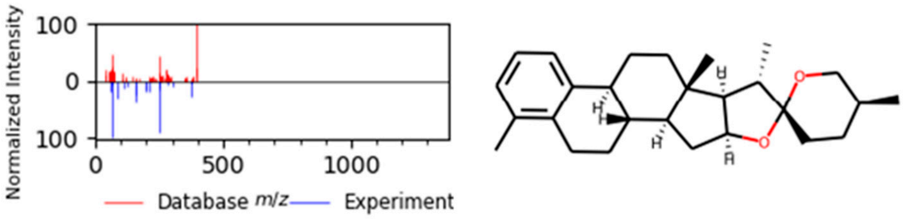

Figure S12. MS/MS spectra of feature with *m/z* 407.331 in cluster 21 (Experiment) compared to spectra from the *in silico* proposed standard UNPD133062 (ISDB database, *m/z* 395.294), and the *in silico* structure (©GNPS). Cosine score as analogs was 0.53.
